# Recommendations for the Support of Clinical Facilitators' Mental Well‐Being in Undergraduate Health Professions Education: A Scoping Review

**DOI:** 10.1111/jnu.70083

**Published:** 2026-04-01

**Authors:** Lizemari Hugo, Antoinette Du Preez, Ronelle Jansen, Marcel Fladimar Mather

**Affiliations:** ^1^ School of Nursing, Faculty of Health Sciences, University of the Free State Bloemfontein South Africa; ^2^ NuMIQ Research Focus Area, School of Nursing, Faculty of Health Sciences, North‐West University, Potchefstroom Campus Potchefstroom South Africa

**Keywords:** clinical facilitators, mental well‐being, nursing, undergraduate students

## Abstract

**Introduction:**

The multifaceted responsibilities borne by clinical facilitators can impose a significant strain on their mental and emotional health and well‐being. Within nursing and other healthcare professions, the responsibilities of clinical facilitators extend beyond conventional clinical duties to encompass supervising, facilitating, assessing, and supporting students as they transition into professional nursing practice. There is a need for insights into the well‐being of clinical facilitators in the face of demanding work conditions, limited resources, and the emotional toll of patient care. This article critically reviews the literature to determine the current state of mental well‐being among clinical facilitators in undergraduate health professions education and how they should be supported.

**Method:**

A scoping review guided the study using the Population, Context, Concept framework with a search string incorporating associated Boolean operators. 233 records were retrieved from eight databases and reviewed according to the inclusion and exclusion criteria. A total of 13 full‐text articles were found to be eligible for extraction and analysis.

**Results:**

Major findings resulted in thematising mental health issue characteristics according to high and low job demands and burnout. Literature outlined the causes of mental health issues, including lack of recognition and appreciation, work performance, teaching and learning practices, student characteristics, support structures, and the environment. Recommendations included training, appropriate support systems, workload, guidelines, monitoring, community of practice, and research‐oriented support.

**Conclusion:**

Educational institutions must move beyond ad hoc support and implement integrated, strategic initiatives grounded in the principles of the Job‐Demand‐Control model. Future research should focus on developing and rigorously evaluating multifaceted intervention programmes that address these systemic factors. By investing in the well‐being of clinical facilitators, institutions ultimately invest in the quality of future healthcare providers and the safety of the patients they will serve.

**Clinical Relevance:**

This article sets the tone for factors to consider and provides recommendations for educational institutions to better support the mental well‐being of clinical facilitators.

## 1 Introduction

Clinical facilitators in nursing and other healthcare professions often face increased workloads as they contribute to both patient care and students' learning. While student support needs have garnered considerable attention over the past decade, comparatively less focus has been given to clinical facilitators who are fundamental to supporting students' learning within complex clinical learning environments. The clinical learning environment is fundamental for the work‐integrated learning of undergraduate students, affording opportunities to apply theoretical knowledge, develop practical skills, and cultivate professional behaviors essential for their future practice (Griffiths et al. 2022; Berndtsson et al. 2020). Within this context, clinical facilitators occupy an irreplaceable role, as undergraduate students in health professions are at a foundational phase and need to acquire skills, integrate theory and practice, while building confidence and being socialized in their respective professions (Vellem and Jooste 2025). Therefore, it is essential for clinical facilitators to offer guidance, support, and feedback as students navigate complex clinical scenarios (Hugo‐Van Dyk, Nyoni, et al. 2022). As facilitators of learning, they exemplify effective decision‐making, patient advocacy, and ethical conduct (Alshareef and Flemban 2025). Through these roles, clinical facilitators facilitate the transformation of academic learning into practical expertise, while ensuring that the clinical learning environment remains conducive to high‐quality patient care (Griffiths et al. 2022). For this review, clinical facilitators will be defined as any healthcare professional who supports students' learning in the clinical learning environment, including but not limited to preceptors, clinical instructors, or clinical teachers.

Clinical facilitators' roles and responsibilities extend far beyond standard patient care to incorporate various supporting strategies, such as cognitive, emotional, and systems support, to aid the transition from undergraduate to professional nursing practice (Hugo‐van Dyk, Nyoni, et al. 2022). This dual role requires facilitators to ensure safe, competent patient care while concurrently educating future nurses, placing them in a position of significant responsibility and psychological demand (Griffiths et al. 2022; Aparício and Nicholson 2020; Barker and Pittman 2010). However, the demanding responsibilities have a significant impact on the mental and emotional health and well‐being of clinical facilitators, especially when confronted with adverse work conditions, limited resources, and patient care (Griffiths et al. 2022; Park et al. 2019; Barker and Pittman 2010).

Recently, there has been increasing attention to the mental health needs of nursing students (Zhu et al. 2021). However, comparatively less consideration has been given to the well‐being of healthcare professionals responsible for supporting their clinical learning. Literature reveals that facilitators frequently encounter high stress due to time constraints, conflicting institutional demands, understaffing, demands from critical care patients, and the emotional labour required to support students in dynamic and often unpredictable clinical environments (Benny et al. 2024; Durkin et al. 2022). The mental toll can be exacerbated by limited institutional support, lack of formal training, including training in educational strategies, and the absence of structured psychological debriefing or peer support mechanisms (Doran 2024; Durkin et al. 2022).

The Job Demand‐Control (JDC) Model, developed by Karasek Jr (1979), is a widely used framework for understanding occupational stress and burnout. It posits that the interaction between job demands and job control determines the level of psychological strain experienced by workers. When job demands are high and job control is low, individuals are more likely to experience stress‐related outcomes, including burnout. JDC highlights how high work demands combined with low autonomy, known as ‘high strain’ roles, can severely impact employee mental health and increase burnout risk (De Hert 2020; Zou et al. 2022). For clinical facilitators, intense workloads, time pressures, and complex tasks often heighten stress levels.

Emerging literature suggests that systemic interventions can alleviate psychological strain for clinical facilitators and protect their mental well‐being (Doran 2024). Evidence‐based strategies, such as structured clinical facilitator training programmes that integrate clinical skills development with pedagogical knowledge and resilience, can enhance clinical facilitators' confidence and competence in their teaching roles (Skoglund et al. 2023). Offering facilitators more decision‐making power and control over schedules and teaching methods helps buffer the negative impacts. Enhancing job control, even if workload stays the same, may be a key strategy for improving well‐being and preventing burnout in clinical education.

Without adequate interventions, these stressors may culminate in emotional exhaustion, mental fatigue, and ultimately burnout (Aparício and Nicholson 2020). Burnout is conceptualized as a multidimensional syndrome encompassing emotional, physical, and mental exhaustion (De Hert 2020; Hillert et al. 2020; Maslach and Leiter 2016). It is defined as a psychological condition arising from prolonged exposure to persistent interpersonal stressors encountered in the workplace (Maslach and Leiter 2016). Burnout can lead to negative individual, situational, and organizational outcomes (Maslach and Leiter 2016).

Such outcomes not only compromise the quality of clinical facilitation but can also impair patient care and discourage nurses from continuing in the clinical facilitator role (Ogbeide et al. 2023). Given that the clinical education model heavily relies on the availability and effectiveness of clinical facilitators, it is crucial to prioritize their mental health. Despite the growing body of evidence supporting these interventions, many healthcare institutions fail to implement them consistently, resulting in significant variations in the support available to clinical facilitators. This inconsistency highlights the pressing need for a comprehensive, system‐wide approach to improving the mental well‐being of facilitators.

Ensuring the mental health and well‐being of clinical facilitators is crucial for maintaining the quality and sustainability of clinical nursing education. Clinical facilitators encounter distinct challenges that can have a considerable psychological impact, including role overload, inadequate training, insufficient institutional support, and emotional fatigue (Doran 2024; Durkin et al. 2022). By instituting structured support systems and providing access to resources, healthcare institutions can alleviate the psychological burden experienced by clinical facilitators and promote their mental well‐being. However, there is limited research done on the mental well‐being of clinical facilitators and the support needed for them to prepare a future‐ready workforce. Therefore, this review aimed to map the existing literature concerning the mental well‐being of clinical facilitators involved in undergraduate health professions education and how they should be supported. Recommendations from this review can be used by institutions and included in models to better support clinical facilitators' well‐being while supporting students' learning in clinical practice.

## 2 Material and Methods

### 2.1 Design

A scoping review was guided by the methodological framework outlined in the Joanna Briggs Institute (JBI) Manual for Evidence Synthesis (Peters et al. 2020), providing a structured and systematic approach to evidence mapping and synthesis. A systematic approach was used as stipulated by the JBI framework by (1) defining the review question, (2) developing inclusion and exclusion criteria, (3) describing the search strategy, (4) selecting sources of evidence, (5) data extraction, (6) analyzing the evidence and presenting the results (Aromataris et al. 2024). The primary objective was to explore the existing literature on the mental well‐being of clinical facilitators in undergraduate health professions education and to determine how they should be supported. The protocol was registered at the Open Science Framework (see https://osf.io/k4caz/). The study was approved by the first author's institutional ethics committee: UFS‐HSD2025/1833/2810.

### 2.2 Defining the Review Question

The Population (clinical facilitators), Concept (mental well‐being), Context (undergraduate health professions education) (PCC) framework was used to structure the review question:

What is the current state of the literature on the mental health of clinical facilitators in undergraduate health professions education, and how should they be supported?

### 2.3 Inclusion and Exclusion Criteria

The PCC framework informed the development of inclusion and exclusion criteria, as well as the literature search on the mental well‐being of clinical facilitators in undergraduate health profession education. Mental health nursing and psychiatric nursing were excluded to focus the search on relevant sources. Literature containing postgraduate students or students from other professions was excluded, as well as articles unrelated to mental health or well‐being.

However, studies that included both newly qualified personnel, such as newly qualified nurses, and students as a total population were included. No data limiters were applied, as researchers aimed to seek the breadth of the literature. Peer‐reviewed articles published in English were included. The first author contacted corresponding authors when articles were published in languages other than English; if English versions could not be sourced, they were excluded from the review. The authors decided to exclude gray literature as it may lack peer review, quality, methodological rigor, and make data extraction difficult. The search was conducted up to April 2025, with no date limiters.

### 2.4 Search Strategy and Information Source

The authors developed a search strategy to identify relevant literature across multiple databases. The formulation of the search string was done using a combination of keywords and Medical Subject Headings (MeSH) terms aligned with the PCC framework. Boolean operators (AND, OR) were employed to ensure sensitivity and specificity. The following search string was used: (“clinical facilitator*” or preceptor* or “clinical mentor*” or “clinical teacher*” or “clinical educator*” or “clinical accompanist*” or “clinical instructor*” or “clinical supervisor*”) n5 (“mental* health*” or “mental* well*” or “mental condition*” or “mental* ill*” or depress* or anxiety or burnout or “mental hygiene” or “mental state” or “psychological condition*” or “psychological state*”) (train* or teach* or educat*) n4 (“health* science*” or health care science* or “health* profession*” or health care profession* or dietetic* or paramedic* or medical or medicine or nurs* or physiotherap* or “physical therap*” or optometr* or “occupational therap*” or dentist* or biogenetic* or radiograph* or biokinetic*) not (mental health nursing or “psychiatric nursing”).

Eight databases were used for the search, including Academic Search, APA PsycINFO, CINAHL, Education Source, Health Source: Nursing Edition, MasterFILE, MEDLINE, and Sociology Source.

### 2.5 Source of Evidence Selection

**FIGURE 1:**
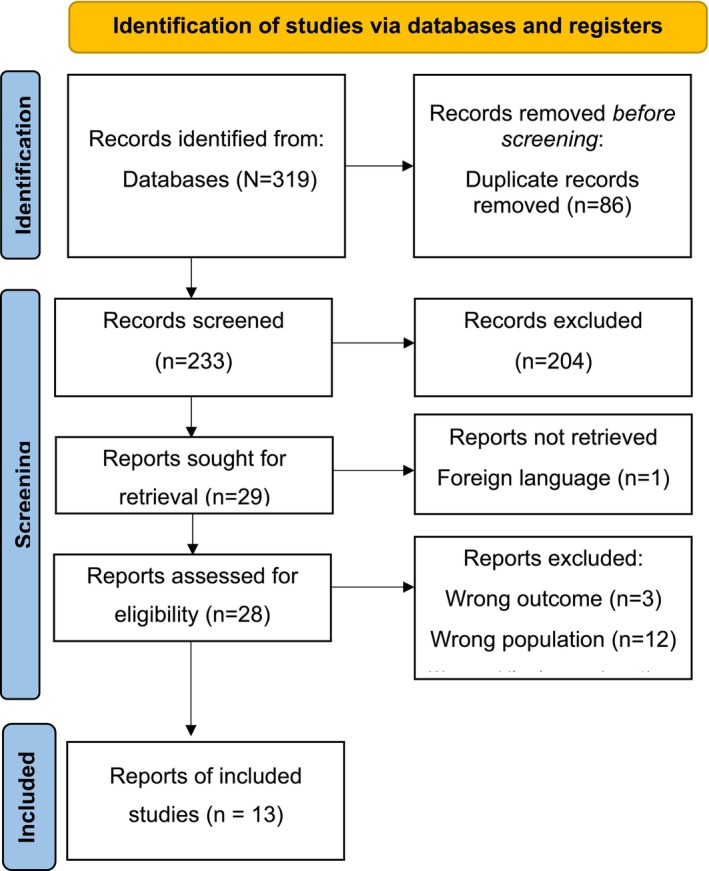
Prisma flow diagram.

A total of 319 records were obtained from the search. The study selection process was facilitated using the Rayyan QCRI platform, a web‐based tool designed for systematic and scoping reviews. After removing duplicates (*n* = 86), titles and abstracts of the 233 records were independently screened by all four authors. Twenty‐eight full‐text reports were subsequently assessed for eligibility by the authors, with discrepancies resolved through discussion and consensus. The PRISMA flow diagram in Figure 1 outlines the selection process, detailing the number of records identified, screened, assessed for eligibility, and ultimately included. Thirteen reports were ultimately included.

### 2.6 Data Extraction

A self‐developed, Excel‐standardized data‐charting form was used and piloted prior to the main data extraction. For the pilot, each author extracted information from three similar articles to ensure that all relevant information was included and to maintain consistency in data extraction. The following information was captured from each included study: citation details, study design, study aim, sample, profession, characteristics of mental health issues, causes of mental health issues, support strategies/interventions/programmes identified, recommendations, study gaps, theories, frameworks, and models. This process enabled a structured synthesis of the literature.

### 2.7 Analysis of the Evidence and Presentation of Results

Information from the full‐text articles was extracted on an Excel spreadsheet as a chart by L.H. and reviewed by R.J. to enhance the reliability of the extracted data. The two authors then individually reviewed the extracted data to enhance rigor.

The authors quantified the study characteristics through descriptive numerical analysis by assigning counts to year of study, sample size, study design, country, and profession. A narrative approach was used to inductively code recurring themes related to burnout, mental well‐being, and support strategies for clinical facilitators, as identified during piloting.

## 3 Results

### 3.1 Characteristics of Included Studies

This review included a total of 13 research articles, as depicted in Table 1. The articles were published between 2002 and 2025, with the majority being published since 2021. Geographically, most studies (*n* = 10) were published in the USA. Four study designs were used. There were eight quantitative (*n* = 8) and three qualitative (*n* = 3) studies. One study employed a mixed‐methods approach, and another utilized a multimethod approach that included a literature review, survey, and focus group discussion. Nursing was the most common profession used to investigate the mental health of clinical facilitators in undergraduate programmes (*n* = 7), followed by pharmacy and dietetics (*n* = 2 each). Kellish et al. (2021) used the term ‘health sciences’ to collectively describe their population without distinguishing professions. The average sample size was about 178 participants, ranging from 494 to 4 in the included studies.

**TABLE 1:** Summary of studies included.

Author(s)	Year	Sample size	Objective(s)	Study design	Country	Profession
Hopkins et al.	2025	310	To identify factors associated with burnout preceptors attribute specifically to the work of precepting.	Quantitative	USA	Dietetics
Pontiff et al.	2024	494	To investigate the prevalence of stress and burnout among Physical Therapy Clinical Instructors using the Perceived Stress Scale–10 and Oldenburg Burnout Inventory surveys.	Quantitative	USA	Physical therapy
Smith et al.	2024	210	To assess nurse preceptor burnout and examine related factors.	Qualitative	USA	Nursing
Hopkins	2023	355	To determine the prevalence of intern‐related burnout in dietetics preceptors and to identify the predictors of this burnout.	Quantitative	USA	Dietetics
Joseph & Mayer	2023	228	To describe key themes discussed by student pharmacists and preceptors related to recognizing burnout and maintaining well‐being during an experiential assignment.	Qualitative	USA	Pharmacy
Durkin et al.	2022	24	To describe the assessment of contributing factors to preceptor burnout and strategies to address them.	Multimethod	USA	Nursing
Hugo‐Van Dyk et al.	2022b	24	To describe the stress and burnout experienced by preceptors during the COVID‐19 pandemic while accompanying students in the CLE.	Mixed method	South Africa	Nursing
Baumgartner et al.	2022	113	To investigate the incidence of burnout among preceptors for pharmacy students and residents using the MBI‐HSS and to identify predictors for the development of burnout in this population.	Quantitative	USA	Pharmacy
Kellish et al.	2021	75	To examine the prevalence of moral injury in clinical educators to determine if a relationship exists between moral distress, burnout, and their roles as clinical educators.	Quantitative	USA	Health Science Clinicians
Hautala et al.	2007	65	To explore the lived experiences of nurse preceptors working with new nurses	Quantitative	USA	Nursing
Allen	2002	115	To explore the preparation and support needed of practitioners involved with preceptorship in pre‐ and post‐registration mental health nursing, and to identify how such needs may be met.	Qualitative	UK	Nursing
Hrobsky & Kersbergen.	2002	4	To explore preceptors' perceptions regarding unsatisfactory clinical performance of students.	Qualitative	USA	Nursing
Yonge et al.	2002	295	To highlight, from the preceptor's perspective, the nature of stress in the preceptor role and to identify the kind of support that is needed to make the preceptorship experience valuable.	Quantitative	Canada	Nursing

### 3.2 Mental Health Issue Characteristics

In the reviewed studies, clinical facilitators described various characteristics of mental health issues, which are listed in Table 2.

**TABLE 2:** Mental health issue characteristics.

Characteristics	Sources
Burnout	Hopkins et al. 2025; Pontiff et al. 2024; Smith et al. 2024; Hopkins 2023; Joseph and Mayer 2023; Durkin et al. 2022; Hugo‐Van Dyk, Nyoni, et al. 2022; Kellish et al. 2021; Baumgartner et al. 2022
Exhaustion	Durkin et al. 2022; Hugo‐Van Dyk, Nyoni, et al. 2022; Joseph and Mayer 2023; Kellish et al. 2021; Baumgartner et al. 2022
Anxiety	Hugo‐Van Dyk, Nyoni, et al. 2022; Allen 2002; Hrobsky and Kersbergen 2002
Fear	Hugo‐Van Dyk, Nyoni, et al. 2022; Hrobsky and Kersbergen 2002
Stress	Pontiff et al. 2024; Joseph and Mayer 2023; Hautala et al. 2007; Young, 2002
Depersonalisation	Kellish et al. 2021; Baumgartner, 2021
Moral injury and distress	Kellish et al. 2021
Overextended	Kellish et al. 2021
Frustration	Durkin et al. 2022
Physical symptoms	Durkin et al. 2022
Isolation	Durkin et al. 2022
Poor work performance	Joseph and Mayer 2023
Low personal accomplishment	Baumgartner et al. 2022

As displayed in Table 2, most of the studies recognized burnout as a mental health issue experienced by clinical facilitators, followed by exhaustion, which encompasses mental, physical, and/or emotional aspects. Additionally, anxiety, fear, stress (including worry), and depersonalization were also mentioned by more than one author.

### 3.3 Causes for Mental Health Issues

One study (Pontiff et al. 2024) did not report the causes of mental health issues. The other 12 studies identified six factors associated with clinical facilitators' mental health issues during the accompaniment of students, including work performance, teaching and learning practices, student characteristics, a lack of recognition and appreciation, support structures, and the environment.

Studies frequently mention issues surrounding clinical facilitators' work performance. Their schedules influenced clinical facilitators' work performance (Durkin et al. 2022), increased workload (Joseph and Mayer 2023; Durkin et al. 2022; Hautala et al. 2007), and responsibility (Kellish et al. 2021; Hautala et al. 2007; Yonge et al. 2002), creating concern in facilitators (Joseph and Mayer 2023). Durkin et al. (2022) noted that appointed clinical facilitators were often not asked to facilitate or received little notice to do so. Their commitment to the facilitation of students' learning led them to go the extra mile, such as taking work home, being available for students, which led to facilitators spending extra time with students (Joseph and Mayer 2023; Yonge et al. 2002), leading to problems in time management of tasks (Joseph and Mayer 2023; Durkin et al. 2022). Several studies have shown that clinical facilitators are often overburdened with patients and other assignments (Durkin et al. 2022; Hautala et al. 2007), which included critically ill patients (Hautala et al. 2007). Baumgartner (2021) also reported that clinical facilitators often worked up to 40 h per week. In addition, the reported high number of students supervised (Hopkins et al. 2025; Durkin et al. 2022; Kellish et al. 2021) and staff turnover (Durkin et al. 2022) affected their work performance. The increase in documentation and paperwork associated with accompaniment also added to the workload of the clinical facilitators (Durkin et al. 2022). The study by Hugo‐Van Dyk, Nyoni, et al. (2022) highlighted the adverse effect of outbreaks on clinical facilitators during clinical accompaniment, which should also be considered. Yonge et al. (2002) highlighted that the expectations placed on clinical facilitators, including their teaching and learning practices, are often considered unrealistic.

The additional teaching and learning roles of students' clinical facilitators, such as supervision, were often mentioned in relation to their well‐being (Kellish et al. 2021; Hautala et al. 2007). Clinical facilitators had to take responsibility for patient safety as they were answerable for students' actions and possible mistakes (Hautala et al. 2007; Yonge et al. 2002). Students' diverse experiences and year levels necessitated that clinical facilitators adapt their orientation and teaching approaches (Durkin et al. 2022; Hautala et al. 2007). Mismatches of teaching and learning approaches, including personality styles, added to facilitators' burnout (Hopkins et al. 2025; Durkin et al. 2022; Kellish et al. 2021). Hrobsky and Kersbergen (2002) reported that facilitators experienced stress when they knew a student would fail if they reported their observations.

As part of their teaching and learning practices, clinical facilitators had to ensure their knowledge and skills were current to provide clear explanations and answers to questions, while introducing students to policies and procedures in the clinical environment (Durkin et al. 2022; Hautala et al. 2007). Hautala et al. (2007) demonstrated that facilitators often lack confidence in their teaching and learning abilities if their roles and goals are not clearly outlined and if they are not adequately prepared. Allen (2002) added that when facilitators do not know the training programme inside out, it often creates anxiety in clinical facilitators. In some cases, clinical facilitators also had to navigate the learning management system (Durkin et al. 2022). Results showed that clinical facilitators should have a community of practice to share information with one another (Hautala et al. 2007).

Another factor influencing facilitators' well‐being was the characteristics of the students. Various student characteristics contributed to clinical facilitators' mental health issues. Students who were unprepared, inexperienced, or unsuited for certain clinical areas affected facilitators' well‐being (Hautala et al. 2007; Yonge et al. 2002). Several studies have highlighted students' lack of critical thinking and unsatisfactory skill performance, which contribute to stress and burnout (Durkin et al. 2022; Hautala et al. 2007; Hrobsky and Kersbergen 2002; Yonge et al. 2002). Student attitudes, such as being unenthusiastic and not asking questions, add to facilitators' burnout (Hrobsky and Kersbergen 2002). Additionally, Hopkins (2023), Baumgartner (2021), and Durkin referred to the stressors of dealing with difficult and unmotivated students who exhibit challenging and unprofessional behaviors.

Also, many studies reported that clinical facilitators felt they deserved special recognition and appreciation for the extra work they do as clinical facilitators (Hopkins et al. 2025; Smith et al. 2024; Durkin et al. 2022; Baumgartner, 2021; Hautala et al. 2007), which was another factor to consider. Baumgartner (2021) mentioned promotion opportunities, bonuses, earning extra hours, and paid time off as possible incentives for organizations to consider showing special recognition to facilitators.

A lack of support structures was also considered a factor for clinical facilitators. Clinical facilitators reported lacking support from organizations, management, and peers for clinical facilitator programmes (Durkin et al. 2022; Hautala et al. 2007).

Hautala et al. (2007) noted a shortage of human resources for the development of clinical facilitators. Durkin et al. (2022) observed that resources for supporting clinical facilitators were lacking. Lastly, the clinical environment in which facilitators had to operate presented challenges, contributing to anxiety and burnout. Facilitators described the clinical environment as busy and fast‐paced (Hautala et al. 2007; Yonge et al. 2002), often with equipment that was unavailable or malfunctioning. (Durkin et al. 2022).

### 3.4 Implemented Strategies, Interventions, and Programmes

Implemented strategies, interventions, or programmes are essential to support clinical facilitators. However, in the scoped studies, we found only two studies that reported on implemented strategies, interventions, or programmes. Only Joseph and Mayer (2023) and a review by Durkin et al. (2022) mentioned implemented programmes for facilitators.

### 3.5 Theories, Frameworks, and Models

Researchers should consider sound theories, frameworks and models when planning and implementing supporting strategies, interventions, and programmes to support clinical facilitators. Among the studies included, only three incorporated a theory, framework, or model. Joseph and Mayer (2023) included an Introductory Pharmacy Practice Experience (IPPE) model in their study, while Hrobsky and Kersbergen (2002) followed the Moraine Park Technical College Preceptor Model. The theoretical framework of Corley's Moral Distress was used by Kellish et al. (2021).

### 3.6 Recommendations

The reviewed literature highlighted several recommendations for nursing education institutions to consider supporting their clinical facilitators. Six recommendations were conceptualized based on the literature, which include training, appropriate support systems, workload management, guidelines, monitoring, a community of practice, and research‐related recommendations. The importance of training programmes for clinical facilitators was the most mentioned recommendation. Training formats to promote confidence in clinical facilitators include programmes, workshops, orientation, and continuous professional development sessions (Pontiff et al. 2024; Durkin et al. 2022; Hrobsky and Kersbergen 2002).

Suggestions include that existing clinical facilitator training needs to be reviewed and strengthened to foster resilience for clinical facilitators to function optimally (Hopkins 2023; Joseph and Mayer 2023; Hugo‐Van Dyk, Botma, and Raubenheimer 2022). Additionally, Hrobsky and Kersbergen (2002) recommend that these programmes be made mandatory in preparation for student encounters. As part of training, the content highlighted by the studies includes educating clinical facilitators on their roles, outcomes of the programme, effective feedback, listening strategies, learning styles, communication, critical thinking, and strategies for managing difficult or struggling students, such as difficult conversations and conflict management (Pontiff et al. 2024; Hrobsky and Kersbergen 2002). Furthermore, facilitator training should include assessment and the implementation of simulation (Durkin et al. 2022). Durkin et al. (2022) and Yonge et al. (2002) also noted that facilitators' competencies should be continuously assessed to determine their readiness.

Support systems were also highlighted as a recommendation. Support will be needed from various systems to enable clinical staff to perform optimally (Hrobsky and Kersbergen 2002; Yonge et al. 2002). Support structures include nursing management, onsite coordinators, educators and faculty (Pontiff et al. 2024; Hautala et al. 2007). Hrobsky and Kersbergen (2002) suggest that collaborative relationships and dialogues between facilitators and faculty should be encouraged to establish a liaison relationship, thereby developing protocols, articulating expectations, and discussing clinical outcomes with stakeholders. The study by Durkin et al. (2022) and Yonge et al. (2002) highlighted requests for frequent meetings with management structures, especially during early consultation. During this constructive and respectful feedback, interrelated concerns should be communicated between management structures (Hopkins et al. 2025; Durkin et al. 2022). Yonge et al. (2002) further highlights the importance of on‐site managers in mitigating potential problematic situations. Additionally, facilitators need to be valued, acknowledged, and recognized by these structures for the services they render (Pontiff et al. 2024; Smith et al. 2024; Durkin et al. 2022).

Given the additional demands on clinical facilitators, it is recommended that processes and procedures be implemented to manage their workload. Examples include providing clinical facilitators with a choice to facilitate or offering them breaks between students (Durkin et al. 2022).

Studies recommend adjusting the workload of facilitators to allocate more time for student learning (Hautala et al. 2007; Yonge et al. 2002). Durkin et al. (2022) suggested giving clinical facilitators no patient assignments, or when assignments are given, that facilitators have dedicated time for teaching or autonomy with patient care that meets students' needs. Based on our study, we recommend that guidelines be established for clinical facilitators to support students optimally. Pontiff et al. (2024) and Darkin et al. (2022) highlight that organizations must have guidelines, manuals, checklists, and reference guides available on educational strategies, goals and roles of clinical facilitators to guide their practice and the criteria that constitute a clinical facilitator. During adverse events, Hugo‐Van Dyk, Nyoni, et al. (2022) suggest that processes be put in place to access credible sources and information. Furthermore, clinical facilitators should be carefully selected, and a clear process and criteria should be in place for their selection (Durkin et al. 2022; Yonge et al. 2002).

Additionally, orientation and other documentation should be standardized (Durkin et al. 2022). Furthermore, it is recommended that clinical facilitators be monitored for signs and symptoms of fear, stress, and burnout resulting from work or personal situations, to design effective coping strategies (Pontiff et al. 2024; Hugo‐Van Dyk, Nyoni, et al. 2022; Yonge et al. 2002). Hopkins et al. (2025) emphasized that burnout prevention is key and that strategies should be implemented to enhance the well‐being and retention of clinical facilitators. The last recommendation speaks to a community of practice. Clinical facilitators should have opportunities to share their experiences and voice their challenges through a community of practice (Pontiff et al. 2024; Durkin et al. 2022). Forming a community of practice can also enable facilitators to hand over students to one another (Durkin et al. 2022).

## 4 Discussion

This scoping review synthesizes existing literature on the mental well‐being of clinical facilitators in undergraduate health professions education, revealing a complex interplay of systemic, organizational, and individual factors that contribute to psychological strain and burnout. The findings consistently demonstrate that clinical facilitators operate within a high‐strain environment, as defined by the Job Demand‐Control (JDC) model (Karasek Jr 1979), characterized by excessive job demands and insufficient job control.\n.

The recurrent themes of emotional exhaustion, mental fatigue, and depersonalization underscore the urgent need for structured, multi‐faceted interventions to safeguard clinical facilitator well‐being, which is inextricably linked to the quality of clinical education and, ultimately, patient care. The primary drivers of clinical facilitator burnout identified in this review, namely, excessive workload, inadequate preparation, and insufficient institutional support, align with the core tenets of the JDC model. Clinical facilitators are tasked with the dual burden of maintaining high‐quality patient care while simultaneously fulfilling demanding educational roles, often without a commensurate reduction in clinical responsibilities or an increase in autonomy (Hopkins et al. 2025; Johnson 2020). This role overload is exacerbated by a lack of formal training in pedagogical methods, leaving many clinical facilitators feeling ill‐equipped for their educational duties and contributing to feelings of anxiety and inadequacy (Hilty et al. 2022; Allen 2002). Using sound theories, frameworks, and models enables educators with a structured approach to support strategies and training that are built on existing knowledge. Porat‐Dahlerbruch et al. (2022) state that using formal theories, frameworks, and models helps eliminate biases that may occur during strategy design and implementation process.

The finding that facilitators frequently feel undervalued and unrecognized for their extra labor further compounds this strain, which is a significant demotivator and contributor to emotional exhaustion (Smith et al. 2024; Baumgartner et al. 2022). A critical insight from this review is the profound impact of organizational culture and systemic support structures, or the lack thereof. The absence of clear guidelines, standardized processes, and accessible resources places clinical facilitators in a precarious position, forcing them to navigate complex educational and ethical challenges, such as failing a student or managing unprofessional behavior, with minimal guidance (Hilty et al. 2022). This lack of support not only heightens job demands but also severely limits job control, a key predictor of burnout according to the JDC model.

Poor well‐being of the clinical facilitator with burnout syndrome, according to the Maslach Burnout Inventory Human Service Survey (MBI‐HSS), is characterized by poor decision‐making, fatigue, and a cynical attitude, leading to withdrawal from patients and co‐workers, resulting in increased medical errors (Baumgartner et al. 2022). The COVID‐19 pandemic, as highlighted by Hugo‐Van Dyk, Nyoni, et al. (2022), acted as a magnifying glass, intensifying these pre‐existing systemic vulnerabilities and exposing the critical need for resilient support systems that can withstand healthcare crises.

The recommendations derived from the literature point toward a holistic, institution‐led approach to mitigating burnout. The consistent call for mandatory, ongoing training programs that extend beyond clinical skills to encompass pedagogical strategies, conflict management, and resilience‐building is paramount (Pontiff et al. 2024; Van den Broeck et al. 2017). However, training alone is insufficient. Structural interventions are essential. These include implementing workload adjustments, such as providing dedicated teaching time and reducing patient assignments (Johnson 2020), as well as establishing formal recognition and reward systems to validate clinical facilitator contributions (Baumgartner et al. 2022). Furthermore, fostering communities of practice can mitigate isolation by creating formal channels for peer support, shared problem‐solving, and psychological debriefing (Pontiff et al. 2024; Johnson 2020).

Despite these clear recommendations, a significant gap persists between evidence and implementation. Many included studies noted a lack of formally evaluated interventions and programs, indicating that most healthcare institutions have yet to adopt a systematic, evidence‐based approach to supporting the well‐being of clinical facilitators (Van den Broeck et al. 2017; Johnson 2020). This implementation gap underscores a fundamental disconnect between the acknowledged importance of clinical facilitators and the allocation of resources necessary to support them.

### 4.1 Limitations of the Review

As with all studies, this scoping review has several limitations. A small sample of studies was included. Gray literature was excluded, which could have added to the findings in the review. The predominance of studies from the USA and within the nursing profession may limit the generalizability of the findings to other geographical contexts and health disciplines. Furthermore, the reliance on self‐reported data in many of the included studies introduces the potential for response bias. The methodological limitations of the primary studies, such as small sample sizes and a lack of longitudinal designs, as noted in the gaps, prevent causal inferences about the effectiveness of proposed interventions. We acknowledge that the quality of the included studies and potential bias can influence the reported outcomes and impact the robustness of the overall conclusion.

## 5 Conclusion

The mental well‐being of clinical facilitators is a critical component of a sustainable and effective clinical education system. Protecting this valuable resource requires a paradigm shift from viewing clinical facilitators as an individual responsibility to recognizing it as an institutional imperative. Healthcare and educational institutions must move beyond ad‐hoc support strategies and implement integrated, strategic initiatives founded on sound frameworks. This involves proactively reducing job strain by enhancing clinical facilitator control over their work and providing them with the support, training time, and recognition needed to succeed. Future research should focus on developing and rigorously evaluating multifaceted intervention programs that address these systemic factors, particularly in under‐researched geographical regions and health professions. Healthcare education institutions can use the information provided in this article to promote the well‐being and support of clinical facilitators, ultimately investing in the quality of future healthcare providers and the safety of the patients they will serve.

## Conflicts of Interest

The authors declare no conflicts of interest.

## Data Availability

The data that support the findings of this study are available from the corresponding author upon reasonable request.
